# TF-ChIP Method for Tissue-Specific Gene Targets

**DOI:** 10.3389/fncel.2019.00095

**Published:** 2019-03-19

**Authors:** Amalia Perna, Lavinia Auber Alberi

**Affiliations:** ^1^Department of Medicine, University of Fribourg, Fribourg, Switzerland; ^2^Swiss Integrative Center for Human Health, Fribourg, Switzerland

**Keywords:** ChIP, transcription factor, Notch signaling, brain, low amount DNA, region-specific, gene expression

## Abstract

Chromatin immunoprecipitation (ChIP) is an assay developed in order to define the dynamic nature of transcription processes. This method has been widely employed to identify methylated and acetylated DNA sequences in a variety of organs both in animals and humans. Nevertheless, this technique is significantly less employed to study transcriptional targets of specific nuclear signaling factors (TFs) and the data published so far have mainly used cell culture material and have been hardly reproduced in *ex-vivo* tissue. As nuclear signaling underlies important adaptive and maladaptive responses in chronic conditions such as cancer and neurodegeneration, there is a need for streamlining the upfront workflow of TF-ChIP for subsequent target sequencing. Based on the typical low concentration of the signaling transcriptional complex and the complexity/length of the ChIP Seq protocol, the field of cellular signaling has been confronted with a major roadblock in identifying clinically relevant targets of pathological and physiological signaling pathways. The present protocol offers a standardized procedure for detecting signaling targets in any whole tissue or specific dissected regions. The advantages of the protocol compared to the existing published methods are: (1) the small amount of starting material; appropriate for tissue subregions; (2) the optimization of DNA fragmentation from whole tissue; (3) suitability for sparsely populated tissues (i.e., brain); (4) the specificity of the TF-targeting readout; and (5) high DNA quality for sequencing or hybridization. The present protocol is highly detailed and can be reproduced using both fresh and fresh-frozen tissue. This is particularly relevant in the clinical setting, where specimen integrity is often the limiting step and where transcriptional target profiling is therapeutically relevant. The method is centered on Notch signaling but can be applied to a variety of nuclear signaling pathways as long as specific antibodies are available for pull down. Taken the superior yield/readout of this procedure, ChIP may finally provide relevant information about dynamic downstream gene changes *in vivo* for use in both basic research and clinical applications.

## Introduction

Control of gene expression is essential for a cell to assemble the gene products it needs to regulate cellular homeostasis and communication. Virtually, any step of gene expression can be modulated, from the DNA–RNA transcription step (Genuth and Barna, 2018) to transcripts triaging through miRNA, transcript splicing, protein translation through initiation complex, and post-translational modification of a protein (Mansuy and Mohanna, 2011). The various checkpoints of gene expression warrant a finely tuned biological repertoire determining the cellular identity in a context-dependent manner. Direct interaction of a protein with DNA is the simplest and the most direct method by which a protein changes transcription levels conferring the appropriate fitness in response to environmental changes (Olfson and Ross, 2017). In the setting of neurons, transcriptional control is often activity dependent and induces the expression of genes regulating synaptic plasticity and neuronal network function (Flavell and Greenberg, 2008; Engelmann and Haenold, 2016). Under this light, the transcription factor (TF) assumes high relevance. By binding to a specific DNA sequence, a TF controls the rate of transcription of genetic information and it represents the last wheel in the gear chain of a signaling pathway, determining the pathways functional outcome. Nonetheless, most TFs do not work alone. Many large TF families form complex homotypic or heterotypic interactions through dimerization and others may recruit intermediary proteins such as cofactors that modulate the effects of TFs by promoting (activator) or blocking (repressor) the recruitment of RNA polymerase to specific genes and their transcription (Vaquerizas et al., 2009).

Furthermore, despite transcriptional processes are highly conserved, human tissues differ from other mammalian species by gene expression patterns thereby adding another layer of complexity (Qian et al., 2005; Huang et al., 2018).

One of the techniques that has best helped understanding transcriptional factors’ biological relevance is chromatin immunoprecipitation (ChIP). ChIP is a powerful technique used to detect, map, and quantify the contacts established between the protein of interest and genomic DNA *in vivo*.

As its name suggests, ChIP relies on the use of antibodies to precipitate or pull-down a DNA-binding protein of interest bound to its genomic locus. These DNA fragments are, then, isolated and characterized in order to draw conclusions about a certain transcriptional process.

Despite a simple working principle, ChIP is notoriously technically challenging, highly sensitive to operational errors and artifacts, and often requires a great deal of upfront optimization before reliable results can be obtained. Moreover, it is expensive and time-consuming.

Here, we present a protocol optimized for TF in brain tissues whose extraordinary complexity is thought to rely on refined transcriptional networks and hierarchies.

In particular, we choose to perform this protocol in the Cornus Ammonis (CA) field of the hippocampus (comparing it with cortex) in order to demonstrate the validity of the technique for a little amount of region-specific starting material and provide a best practice TF-ChIP method for researchers working with whole animal and human tissue.

We decided to focus on Notch1 signaling pathway since it provides a good example for low complexity pathway studies. Notch signaling is a well-known player in the embryonic development but increasing evidence points out a critical role in the mature brain of vertebrates and invertebrates. This pathway appears to be involved in neural progenitor regulation, neuronal connectivity, synaptic plasticity, and learning/memory (Alberi et al., 2013). In addition, Notch was also found aberrantly regulated in neurodegenerative diseases, including Alzheimer’s disease and ischemic injury both in rodent models and human tissue (Brai et al., 2016; Marathe et al., 2017) but the mechanistic implication of Notch-dependent targets in this setting remains largely unraveled, limiting its clinical relevance. The signaling is composed by a downstream transcriptional factor, recombining binding protein suppressor of hairless (RBPjK) that normally behaves as a repressor and that needs another protein, Notch intracellular domain (NICD), to be activated and transcribe its target genes. Notch1 signaling has a sophisticated program of gene expression orchestrated by the alternating status of RBPjK. Notch1 is a transmembrane receptor scattered across the cell membrane. Ligand proteins bind to the extracellular domain inducing proteolytic cleavage and release the intracellular domain (NICD), which enters in the nucleus and binds RBPjK. This allows transcription and the subsequent cell response (Siebel and Lendahl, 2017).

The ChIP assay executed in this manuscript is performed specifically in the hippocampal CA fields and involves NICD precipitation in order to assess target genes downstream Notch1 canonical activation.

This work aims to explain in detail the critical steps of the procedure which can be further adapted to any experimental setting using *ex-vivo* tissue (tissues with high extracellular matrix density, secondary factor precipitation, low chromatin yield, signaling pathways with few target genes, etc.) in a very short time.

## Materials and Equipment

### Animals

All experiments on mice were performed with permission of the local animal care committee (Canton of Fribourg, Switzerland approved the Protocol no. 2016_32_FR) and according to the present Swiss law and the European Communities Council Directive of 24 November 1986 (86/609/EEC). C57Bl6 male mice were used throughout the experiment. Transgenic Notch Reporter [Tg(C-EGFP) 25 Gaia/Reya; Jax, FL, United States] was used for fluorescent immunohistochemistry (IHC). All animals (3 months of age) were housed on 12 h light–dark cycle with access to food and water *ad libitum*. Mice were euthanized using pentobarbital (120 mg/kg). Once no pinch reflex was assessed, animals were perfused using 0.9% NaCl and either dissected in ice cold saline solution under a stereoscope (Leica, Germany) or immersion fixed using paraformaldehyde (Sigma–Aldrich, United States; P6148).

### ChIP Equipment

Glass dish

Razor blades

Pestle

Eppendorf

Pippettor

Rotating wheel

Prism R^TM^ Refrigerated MicroCentrifuge (Labnet, Switzerland; C2500-R)

Vortex-Genie 2 (Scientific Industries, Inc., United States; SI-0236)

Bioruptor Plus (Diagenode, Belgium; B01020001)

1.5 ml Bioruptor^®^ Plus TPX microtubes (Diagenode, Belgium; C30010010-300)

Qubit Fluorometric Quantitation (Thermo Fisher, United States; Q33226)

ThermoMixer^®^ C (Eppendorf, Germany; #5382000015)

Mupid^®^-One Electrophoresis System (Eurogentec, Germany)

Omega Lum^TM^ W Imaging System (Aplegen, United States; 81-12120-00)

Biometra TRIO Thermal Cycler Series (Analytik Jena, Germany)

Fragment Analyzer (Advanced Analytical AATI, United States)

Mic qPCR Cycler (Bio Molecular Systems, Australia).

### ChIP Reagents

Formaldehyde solution, 36.5–38% (Sigma-Aldrich, United States; F8775) !caution formaldehyde is toxic by inhalation, ingestion, or contact with skin.

Phosphate-buffered saline (PBS) (10×) pH 7.4 (Thermo Fisher Scientific, United States; AM9625)

Glycine (Sigma–Aldrich, United States; G8898)

Protease inhibitor (PI) cocktail (Sigma–Aldrich, United States; P8340)

Sodium dodecyl sulfate (SDS) (Roth, Germany; CN30.3) !caution SDS is toxic by inhalation, ingestion, or contact with skin.

Ethylenediaminetetraacetic acid (EDTA) (Sigma–Aldrich, United States; E5134)

Tris (Roth, Germany; 5429.3)

4-(2-hydroxyethyl)-1-piperazineethanesulfonic acid (HEPES) (Sigma–Aldrich, United States; H3375)

NaCl (Roth, Germany; 9265.2)

Sodium deoxycholate (Sigma–Aldrich, United States; 30970)

Triton X-100 (Sigma–Aldrich, United States; 93426)

Tris–HCl (Roth, Germany; 9090)

LiCl (Sigma–Aldrich, United States; L9650)

Nonidet P-40 substitute (Sigma–Aldrich, United States; 74385)

Qubit^TM^ dsDNA HS Assay Kit (Thermo Fisher, United States; Q32851)

Pierce^TM^ Protein A/G Agarose (Thermo Fisher, United States; 20421)

Antibodies: Rabbit anti-NICD (Cell Signaling, United States; #4147); Rabbit IgG (Cell Signaling, United States; #2729); and Rabbit anti-Acetyl-Histone H3 (Lys9) (Cell Signaling, United States; #9649)

RNase A solution (Promega, United States; A7974)

Proteinase K (Roche, Switzerland; 03508838103)

Phenol/chloroform/isoamyl alcohol (Roth, Germany; A156) !Caution chloroform is toxic if absorbed through the skin, inhaled or ingested.

Chloroform/isoamyl alcohol (Roth, Germany; X984) !Caution chloroform is toxic if absorbed through the skin, inhaled or ingested.

Sodium acetate (Merck, Germany; 1.06268.0250)

Glycogen (Roche;10901393001)

Isopropanol (Fisher Chemical, United States; P/7500/15)

Ethanol absolute (Fisher Chemical, United States; E/0650DF/15)

Agarose standard (Roth, Germany; 3810)

SYBR^TM^ Safe DNA Gel Stain (Thermo Fisher, United States; S33102)

GoTaq^®^ Hot Start Green Master Mix (Promega, United States; M5122)

GoTaq PCR Master mix (Promega United States; A6001)

GeneRuler 100 bp DNA Ladder, ready-to-use (Thermo Fisher, United States; SM0243)

DNA Gel Loading Dye (6×) (Thermo Fisher, United States; R0611)

DNF-474 High Sensitivity NGS Fragment Analysis Kit (1–6,000 bp) (Advanced Analytical, United States).

### Reagent Setup

Before preparing the final solutions, filter both the water and all the stock solutions. Here volumes are calculated for three precipitations: one for the protein of interest (NICD) plus positive (H3) and negative control (IgG). Scale up if more TFs are planned to be used.

1% FA-PBS (fresh/1 ml for tissue): Mix 972 μl of PBS and 28 μl of FA.

Glycine (2 M stock): Solubilize 1.5 g of glycine in 10 ml of Milli-Q water.

Lysis buffer (fresh/1 ml): Mix 30 μl of SDS [10% (vol/vol) stock solution], 20 μl of EDTA (0.5 M stock solution), 50 μl of Tris pH 8.0 (1 M stock solution), 10 μl of PIs, and fill with filtered Milli-Q water to a final volume of 1 ml. Keep on ice until use. Final concentrations: 0.3% SDS, 10 mM EDTA, 50 mM Tris pH 8.0. ! Critical PI must be added just before use.

IP buffer (10× stock/15 ml): Mix 3 ml of HEPES pH 8.0 (1 M stock solution), 6 ml of NaCl (5 M stock solution), 600 μl of EDTA (0.5 M stock solution), and fill with filtered Milli-Q water to a final volume of 15 ml. Final concentrations: 0.2 M HEPES pH 8.0, 2 M NaCl, 0.02 M EDTA. IP stock solution can be stored in the fridge for several months.

IP buffer (1× fresh/3 ml): Mix 300 μl of high salt IP buffer (10× stock), 30 μl of NaDOC [10% (vol/vol) stock solution], 300 μl of Triton X-100 [10% (vol/vol) stock solution], 30 μl of PIs, and fill with filtered Milli-Q water to a final volume of 3 ml. Keep on ice until use. Final concentrations: 1% high salt IP buffer, 0.1% NaDOC, 1% Triton X-100 ! Critical PI must be added just before use.

Washing buffer – reduced SDS (stock/50 ml): Mix 2.5 ml of HEPES pH 7.6 (1 M stock solution), 1.5 ml of NaCl (5 M stock solution), 5 ml of Triton X-100 [10% (vol/vol) stock solution], 500 μl of NaDOC [10% (vol/vol) stock solution], 250 μl of SDS [20% (vol/vol) stock solution], and fill with filtered Milli-Q water to a final volume of 50 ml. Washing solutions can be stored in the fridge for several months. Final concentrations: 50 mM HEPES pH 7.6, 2 mM NaCl, 1% Triton X-100, 0.1% NaDOC, and 0.1% SDS

Washing buffer – reduced SDS buffer + NaCl (stock/15 ml): Mix 15 ml of reduced SDS buffer and 600 μl of NaCl (5 M stock solution). Washing solutions can be stored in the fridge for several months. Final concentrations: 0.2 M NaCl.

Washing buffer – NP-40 buffer (stock/25 ml): Mix 0.2 ml of Tris–HCl pH 8.0 (1 M stock solution), 0.625 ml of LiCl (8 M stock solution), 40 μl of EDTA (0.5 M stock solution), 1 ml of NP-40 [10% (vol/vol) stock solution], 1 ml of NaDOC [10% (vol/vol) stock solution], and fill with filtered Milli-Q water to a final volume of 25 ml. Washing solutions can be stored in the fridge for several months. Final concentrations: 8 mM Tris–HCl pH 8.0, 2 mM LiCl, 0.8 mM EDTA, 0.4% NP-40, and 0.4% NaDOC.

Washing buffer – TE buffer (stock/50 ml): Mix 0.5 ml of Tris–HCl, pH 8 (1 M stock solution), 100 μl of EDTA (0.5 M stock solution), and fill with filtered Milli-Q water to a final volume of 50 ml. Washing solutions can be stored in the fridge for several months. Final concentrations: 10 mM Tris–HCl, pH 8, 1 mM EDTA.

Elution buffer (fresh/2 ml): Mix 100 μl of Tris, pH 8 (1 M stock solution), 40 μl of EDTA (0.5 M stock solution), 100 μl of SDS [20% (vol/vol) stock solution], and fill with filtered Milli-Q water to a final volume of 2 ml. Elution buffer can be stored in the fridge for several months. Final concentrations: 50 mM Tris pH 8, 10 mM EDTA, and 1% SDS.

Protein A- or protein G-sepharose beads (Pierce^TM^ Protein A/G Agarose; Thermo Fisher, United States; 20421).

### Primers List

Primers were obtained from Microsynth, Switzerland.

**Table:** 

	Forward	Reverse
Hes5	5′-GGG AAA AGG CAG CAT ATT GAG GCG-3′	5′-CAC GCT AAA TTG CCT GTG AAT TGG CG-3′
DLL1	5′-GTC TCA GGA CCT TCA CAG TAG-3′	5′-GAG CAA CCT TCT CCG TAG TAG-3′
GAPDH	5′-GAT TAC GGG ATG GGT CTG AA-3′	5′-GCT GCA CCT CTG GTA ACT CC-3′


### Immunohistochemistry

Floating coronal brain sections from the TNR-EGFP mice, exposed to a novel environment (Alberi et al., 2011), were processed for fluorescent IHC according to the previously published protocols (Marathe et al., 2017) using Rabbit anti-NICD (Cell Signaling, United States; #4147) and goat anti-EGFP (Rockland, United States; #600-102-215). Cy3 donkey anti-rabbit (Jackson Immunoresearch, United Kingdom; #711-165-152) and Cy2 donkey anti-goat (Jackson Immunoresearch, United Kingdom; #705-225-147) were used as secondary antibodies. DAPI (Sigma–Aldrich, United States; D9542) was used for nuclear counterstaining.

### Western Blot

Input, unbound, and ChIP-NICD fractions were collected and de-crosslinked by adding 11 μl of 5 M NaCl and 4 μl of RNaseA, and incubating in the thermoshaker for 4 h at 42°C with gentle shaking. Proteins were extracted with Trizol (Trifast; Peqgold, Germany) and resuspended in loading buffer. Twenty-five microliters of protein was loaded onto a 10% polyacrylamide electrophoretic gel. Western blot was conducted as previously described (Marathe et al., 2017) using two primary antibodies: polyclonal goat anti-Notch1 against the c-terminus, 1:1000 (sc-6014; Santa Cruz Biotechnology, United States) and mouse anti-β-actin, 1:2000 (sc-81178; Santa Cruz Biotechnology, United States); and two secondary antibodies diluted 1:2000: Cy3 donkey anti-goat (Jackson Immunoresearch, United Kingdom; # 705-165-147) and Cy2 donkey anti-mouse ( Jackson Immunoresearch, United Kingdom; # 715-225-150). Visualization was performed through a fluorescent imager (Omega Lum G from Aplegen).

## Procedure

### ChIP Protocol Overview

**FIGURE 1:**
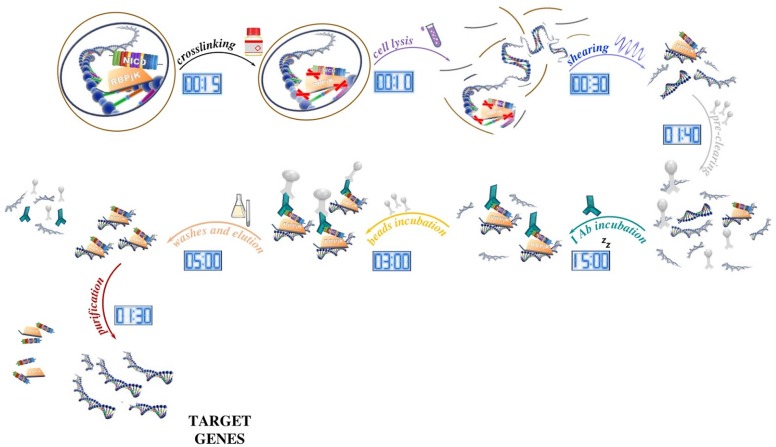
Schematic of the ChIP method and its timing. After tissue processing, DNA and proteins are reversibly crosslinked with short formaldehyde incubation (15 min) to maintain the association of proteins with their target DNA sequence. In our ChIP experiment, NICD and RBPjK are first crosslinked with DNA and then cells lysed with ionic lysis buffer (10 min). Chromatin is later fragmented with ultrasounds (30 min) and precleaned with sepharose beads incubation (1 h 40 min). Once all the unspecific DNA fragments are removed, proteins bound to DNA are ready to be targeted by the selected antibody. After ON incubation, antibody–protein–DNA complexes are precipitated using beads (3 h). Washes and elution (5 h) lead to target genes that once purified (5 h) are ready for further applications.

The protocol can be essentially divided into several phases. During the first step, the sample is treated with formaldehyde to cross-link proteins and DNA within ∼2 Å of each other. As a result, proteins are covalently bound to their target sequences on the DNA and provide a snapshot of TF occupancy. Later, the chromatin fraction is released from the nucleus by the mean of a lysis buffer, fragmented into small pieces (ranging from 200 to 500 bp) with a sonicator, and then subjected to immunoprecipitation with chosen antibodies. Once immune complexes are obtained, they are separated from the unbound fraction by using protein A- or protein G-sepharose beads (magnetic protein A/G beads can be used instead of sepharose beads, however, washes are performed using an appropriate magnetic tray). After extensive washing to limit the nonspecific binding, the immune-bound complexes are eluted, decrosslinked by proteinase K digestion, and then purified in order to be analyzed (Figure 1).

Protein–DNA interactions can, then, be approached fundamentally in two ways: (i) by looking at the presence of specific sequences in the immunoprecipitated DNA by PCR amplification or (ii) by combining genome-wide approaches culminating in a method for deep protein–DNA interactions analysis: ChIP–Seq.

### Considerations Before Starting

• In order to perform a tissue-specific ChIP analysis, it is important to select the specific part of the tissue you are interested in. For peripheral organs, blood could interfere with the purity of your sample, therefore is recommended to transcardially perfuse the animal with saline solution before dissection.
• The tissue could be made of subregions with different cell populations where the signaling behaves differently. It is worth to study *a priori* your tissue anatomy and composition to reduce the background and obtain unequivocal results. In our case, during hippocampus dissection, we carefully removed the dentate gyrus (composed by neuronal progenitor cells and granule cells) and then we selected just the CA field (Sultan, 2013). For the cortex, we removed the white matter to increase the gray matter content for ChIP analysis.
• While planning the experiment, remember to include always a positive control as a very abundant core histone protein (i.e., H3) and a negative control that is a sample treated in the same way as in the immunoprecipitated samples but without Ab or with a non-specific Ab (i.e., IgG).
• For optimal chromatin yield and ChIP results, it is important to use at least 25 mg of tissue for each immunoprecipitation. The chromatin yield does vary between tissue types and some tissues may require more than 25 mg depending on their cell density. Since positive and negative control must be included, at least 75 mg of tissue is required.

### Stepwise Procedure

Crosslinking (timing: ca. 40 min):

Before starting the procedure, prepare all your solutions and keep them refrigerated. Switch on the cold centrifuge and set the Bioruptor sonicator at 4°C. Perform all steps on ice to avoid proteins degradation.

1. After tissue dissection from an anesthetized mouse (Figure 2A), snap-freeze the tissue in liquid nitrogen or, if fresh tissue is required, proceed with the protocol.

**FIGURE 2:**
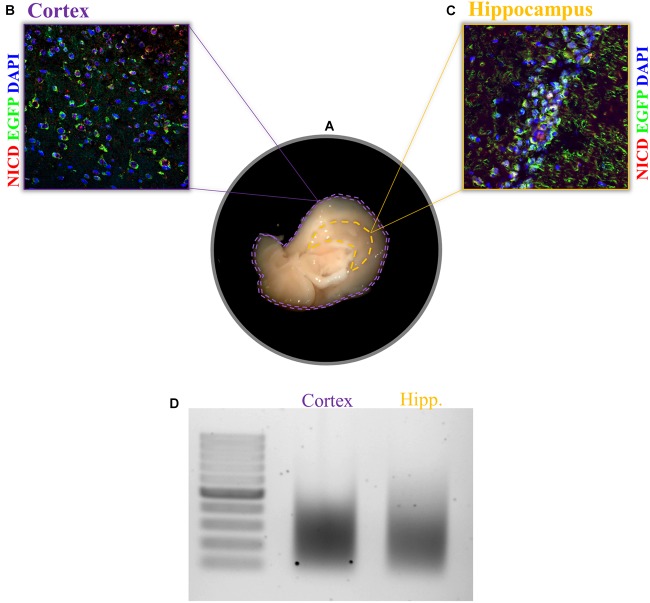
Expression of NICD in cortex and hippocampus and chromatin shearing of the two brain tissues. **(A)** Sagittal brain sections, view from the midline, display the hippocampus and cortex. Example of immunofluorescence for NICD (red) in **(B)** cortex and **(C)** hippocampus of the transgenic mouse line (TNR, for transgenic Notch reporter) expressing enhanced green fluorescent protein (EGFP) in cells with Notch canonical signaling activation. Nuclei are stained in blue. **(D)** CA hippocampal fields and cortical tissue 1% formaldehyde fixed are sonicated for 30 cycles (30″ ON/30″ OFF) with the Bioruptor^®^ PLUS at HIGH power setting. All samples were treated with RNase and Proteinase K prior to gel electrophoresis. Scale bar in **B** and **C** is 25 μm.
2. Transfer quickly the tissue in a glass dish already placed in ice.
3. Rapidly put 40 μl of 1% FA-PBS on your tissue piece (this will fix your tissue while it is thawing), start the timer (already set on 15 min), and finely mince the fresh/thawed tissue with two pre-chilled razor blades for 4 min (until it appears slurry and homogeneous).
4. Add 450 μl of 1% FA-PBS in the dish and transfer the tissue to a 1.5 ml tube. Use other 450 μl 1% FA-PBS to harvest tissue residues from the dish and move it into the tube. Incubate for the remaining 10 min at room temperature (RT) on the rotating wheel (make sure to safe-lock the tubes!).
5. Add 63 μl of glycine 2 M to the 940 μl of 1% FA-PBS (final concentration 0.125 M) to terminate cross-linking. Incubate for 10 min at RT on the rotating wheel (make sure to safe-lock the tubes).
6. Wash three times with cold PBS with freshly added PI:
  a. Add 500 μl of 1× PBS+PI
  b. Invert several times
  c. Spin gently at 4°C for 1 min
  d. Remove supernatant
7. Resuspend the tissue in 600 μl of ice-cold lysis buffer with freshly added PI (first add 200 μl, start to pestle, then add the remaining 400 μl).
8. Smash the tissue with a pestle by going 50 times up and down until the solution is clear and without clumps.
9. Keep the lysate 10 min on ice.
  ! Safe stop. Samples can be stored at -20°C at this point.
    Chromatin sonication (timing ca. 40 min):
    Chromatin needs to be fragmented by sonication into not too short and not too long fragments in order to be precipitated.
    Extensive sonication could most likely hit TF-binding sequences and impair them, while long fragments obtained with short sonication are difficult to precipitate.
    Sonication conditions should be optimized for your experimental settings (see the section “Anticipated Results”). Avoid foaming as this results in a decrease of energy transfer within the solution and will decrease the sonication efficiency.
10. Transfer the lysate in TPX tubes from Diagenode. Prepare three tubes with 200 μl each.
11. Bioruptor Plus (Diagenode, Belgium):
  Power: High
  Runs: 3× 10 cycles
  Interval: 30″ ON/30″ OFF
  Note: Vortex gently and spin down between each run.
  During these 30 min, start washing beads.
12. Centrifuge at 13,500 rpm, 4°C for 10 min.
13. Collect the supernatant and transfer all 600 μl in one 2 ml tube.
  ! Safe stop. Samples can be stored at -20°C at this point.
    Determination of DNA fragment size (timing ca. 5 h):
    Sonication is the key step for a successful ChIP experiment and the optimal sonication settings should be determinate for each experimental tissue. Therefore is a good practice to check DNA fragments on agarose gel first times ChIP is performed.
14. Prepare a mix with 60 μl of elution buffer, 4.8 μl of 5 M NaCl, 2 μl RNase A (10 mg/ml), and 40 μl of chromatin. Incubate while shaking at 65°C for 1 h.
15. Add 4 μl proteinase K and incubate 3–4 h at 65°C [this incubation could be extended overnight (ON)].
16. Add 100 μl of phenol/chloroform (1:1), agitate vigorously.
17. Centrifuge for 10 min at 13,500 rpm, 4°C.
18. Collect the aqueous phase.
19. Add 100 μl chloroform/isoamyl alcohol (1:1).
20. Centrifuge for 10 min at 13,500 rpm, 4°C.
21. Collect the aqueous phase.
22. Add 2 μL of glycogen (20 mg/mL) and 30 μl of NaCH_3_COOH (3 M).
23. Flick the tube and add 100 μl of isopropanol.
24. Agitate vigorously the tube several times for 1 min.
25. Leave on ice for 10 min or keep in the freezer for 5 min.
26. Centrifuge 20 min at 13,500 rpm, 4°C.
27. Decant supernatant.
28. Wash by adding 500 μl of 75% EtOH. Spin 5 min at 13,500 rpm, 4°C, and decant the supernatant.
29. Air dry and resuspend in 20 μl of ddH_2_O. Incubate several minutes while shaking at 42°C to dissolve DNA.
30. Run purified DNA in a 1.5% agarose gel with a 100 bp DNA marker to determine fragment size.
  Beads washing (timing ca. 15 min):
31. Take the IP buffer 10× from the fridge and make 3 ml of IP buffer 1× and add 20 μl of PI.
32. Transfer 20 μl of beads for preclearing and 40 μl for the IPs. Cut the tip used in order to dispense them. You will have in total two 1.5 ml tubes for preclearing and three for IPs.
33. Wash beads three times with 500 μl PBS: At each step spin down the beads shortly with gentle centrifugation so they don’t break, wait until they settle down, and then aspirated gently the PBS with a pump using a 10 μl tip and going progressively down with the tip.
34. Wash one time with 500 μl IP buffer with PI. Keep preclearing beads on ice and store IP beads still in IP buffer in the fridge.
  Pre-clearing (timing ca. 1 h 40 min):
  Pre-clearing is an optional step but is recommended to reduce non-specific binding to the beads.
  SDS presence in the lysis buffer is important since it enhances lysis and sonication but it also interferes with protein/antibody and antibody/beads binding. Therefore, it has to be diluted up to 0.1%.
35. Add IP buffer to the chromatin in 3:1 proportion (600 μl chromatin + 1200 μl IP buffer)
36. Split the chromatin over the two pre-clearing tubes and add to the 20 μl beads you kept on ice (900 μl each).
37. Incubate for 1–1.5 h @ 4°C on the rotating wheel (make sure to safe-lock the tubes).
38. Spin down the beads from the pre-cleared chromatin, collect the supernatant, and discard the 20 μl beads.
39. Measure the DNA concentration using Qubit.
40. Take 5% of the total volume of pre-cleared chromatin as input material and freeze it.
  ! Safe stop. Samples can be stored at -20°C at this point.
    Immunoprecipitation and washes (timing ca. 16 h):
    The optimal antibody concentration should be calibrated for each antibody as this can improve the signal-to-noise ratio of your experiment (see the section “Anticipated Results”).
41. Divide the chromatin into three tubes (600 μl per tube) and add the antibodies to each pre-cleared chromatin: (1) specific for the TF, NICD; (2) negative control, IgG; and (3) positive control, histone H3.
42. Put the chromatin with antibodies on a rotating wheel to mix ON at 4°C (make sure to safe-lock the tubes!).
43. Transfer the chromatin + antibodies to the Eppendorf with the 40 μl beads you kept on ice/on the fridge.
44. Rotate for 3 h at 4°C on the rotating wheel (make sure to safe-lock the tubes).
45. Spin down the beads and discard the supernatant.
46. Wash three times with the reduced SDS buffer: At each step, spin down the beads shortly with gentle centrifugation so they don’t break, wait until they settle down and then aspirate the buffer with the sucker using a 10 μl tip and going progressively down with the tip.
47. Wash one time with reduced SDS + NaCl buffer.
48. Wash one time with NP-40 buffer.
49. Wash one time with TE buffer.
  Eluting the chromatin (timing ca. 15 min):
  Prepare fresh elution buffer.
50. Add 500 μl of elution buffer to the beads and 470 μl to the 5% input.
51. Incubate for 12 min at 65°C while shaking. Flick the tube vigorously once during this incubation and once the incubation is finished.
52. Centrifuge 13,500 rpm, 4°C for 1 min.
53. Transfer the supernatant to a new tube, discard the beads.
  Reversing the crosslinking (Input and IP-ed Chromatin) (timing ca.4-5 h):
54. Add 11 μl of 5 M NaCl. Flick tubes briefly.
55. Add 4 μl of RNaseA to the eluted chromatin and incubate in the thermoshaker for 1 h at 65°C with gentle shaking.
56. Add 4 μl of proteinase K to the eluted chromatin and incubate in the thermoshaker for 3–4 h at 65°C with gentle shaking (this incubation could be extended ON).
  Extraction and purification (timing ca. 1.5 h):
  Be careful during the whole extraction and work under a chemical hood.
57. Add 500 μl of phenol/chloroform (1:1), agitate vigorously.
58. Centrifuge for 10 min at 13,500 rpm, 4°C.
59. Collect the aqueous phase.
60. Add 500 μl chloroform/isoamyl alcohol (1:1), agitate vigorously.
61. Centrifuge for 10 min at 13,500 rpm, 4°C.
62. Collect the aqueous phase.
63. Add 50 μl of NaCH_3_COOH (3 M).
64. Flick the tube and add 500 μl of isopropanol.
65. Agitate vigorously for 1 min.
66. Keep samples on ice for 10 min or put the samples at -20°C for 5 min.
67. Centrifuge for 20 min at 13,500 rpm, 4°C.
68. Decant the supernatant.
69. Wash by adding 500 μl of 75% EtOH. Spin 5 min at 13,500 rpm, 4°C, and decant the supernatant.
70. Air dry under the hood and resuspend in 10 μl of ddH_2_O. Incubate several minutes while shaking at 42°C to dissolve DNA.
71. Measure your DNA concentration using Qubit.

#### PCR

Primer should be designed bearing in mind that the sequence you precipitated is small and must include the TF-binding sequence. Make sure it is included in your primer design (see the section “Anticipated Results”) (Figure 4B).

72. Prepare your (Master mix) × *n* samples, using for each sample:

- 7.5 μl hot start GoTaq
- 0.5 μl primers (10 μM)
- ddH_2_O up to 15 μl

73. Add at least 1 ng of DNA

74. Use the following ChIP program:

**Table:** 

step	°C	H:mm:ss	Go to
1	94.0	0:02:00	
2	94.0	0:00:30	
3	56.5	0:00:30	
4	72.0	0:00:30	29×→ Step 2
5	72.0	0:10:00	
6	4.0	inf	


75. Run PCR products in 1.3% agarose gel.

#### qPCR

Once a specific amplicon is identified by qualitative PCR, the relative efficiency of chromatin pull down should be evaluated via qPCR (Figure 4C).

The primers used for qPCR are the same as for the PCR.

76. Prepare your (Master mix) × *n* samples, using for each sample:

- 7.5 μl Promega GoTaq qPCR master mix
- 0.5 μl Primers (10 μM)
- ddH_2_O up to 15 μl

77. Add 1 ng of DNA.

78. Run the standard amplification program according to the manufacturer instructions.

## Anticipated Results

### Starting Material Amount and Crosslinking

Before starting, it is always recommended to check the specificity of the antibody and the activity rate of your TF in the tissue of your interest. One possible way to do this is to perform an IHC of your region of interest. This would help establish the range of starting material to use since this is going to influence the entire experiment. For the experiment we performed in this paper, we found a sparse and nuclear pattern of NICD in cortical tissue as well as in the CA fields of the hippocampus (Figure 2B,C). For low concentrations of the TF, as in our case, more chromatin should be used during immunoprecipitation. It is recommended to increase the amount of chromatin rather than the amount of antibody since the latter is going to increase the background. To further confirm the right amount of starting material, it is important to assess the chromatin yield after cell lysis. Each tissue has a different chromatin yield that depends on cell density and tissue composition. Therefore, it is important to measure the DNA concentration with a fluorometric method (Qubit) to evaluate how much tissue and/or lysis buffer is required for the next steps.

Our sample preparation section is well optimized for fresh-frozen samples. In clinical analyses, the vast majority of samples need to be stored at -80°C for later analysis or dispatching to other labs. One of the improvements provided by this protocol is the ongoing fixation while the tissue thaws. This captures chromatin–protein architectural organization in the moment of defrosting and prevents loss of biological content due to cell damage, thus, preserving the integrity of the sample. Furthermore, the mincing step with the razor blades makes fixative penetration uniform and easier and is superior to mechanical treatment that causes cell loss. That said, cross-linking efficiency is empirical and should be tested by modifying either incubation time or formaldehyde concentration prior to performing the whole procedures. Remember that extensive cross-linking may decrease the solubility of any target DNA–protein complex and cause it to be entrapped in the insoluble material removed by sedimentation.

### Chromatin Shearing

Sonication is very sensitive to changes, and its efficiency depends on several factors such as temperature, cell type, and density, volume, SDS concentration, and the extent of cross-linking. For reproducible results, these parameters should be kept constant.

We use the Bioruptor Plus sonicator from Diagenode equipped with a cooling water bath and a tray holding up to six samples. To shear DNA, ultrasonic waves are transferred through water generating heat. With this system, the temperature is kept at 4°C preserving sample antigenicity. This system is suitable for shearing up to six sample at a time, assuring reproducibility between replicates. Samples’ volume, tissue composition, and density impact the sonication outcome. If new tissues are going to be tested with this protocol and there is no previous knowledge of their sonication settings, several optimization steps are required. Samples volumes should be fixed at the beginning and sonication efficiency calibrated by testing several aliquots of the starting material at increasing dilutions in lysis buffer (es. 1×, 2×, 4×, 6×, 8×, 10×). By keeping settings constants, it is possible to retrieve the optimal conditions for your tissue.

We achieved a nice shearing (Figure 2D) using 200 μl of both cortical and hippocampal lysates (0.3% of SDS) with a DNA concentration between 6 and 7 ng/μl performing three runs of 10 cycles each with intervals of 30 s ON and 30 s OFF at high power.

It is important to notice that very low SDS concentration and prolonged crosslinking also impact the shearing efficiency. This should be taken into account if any changes are planned to be made.

**FIGURE 3:**
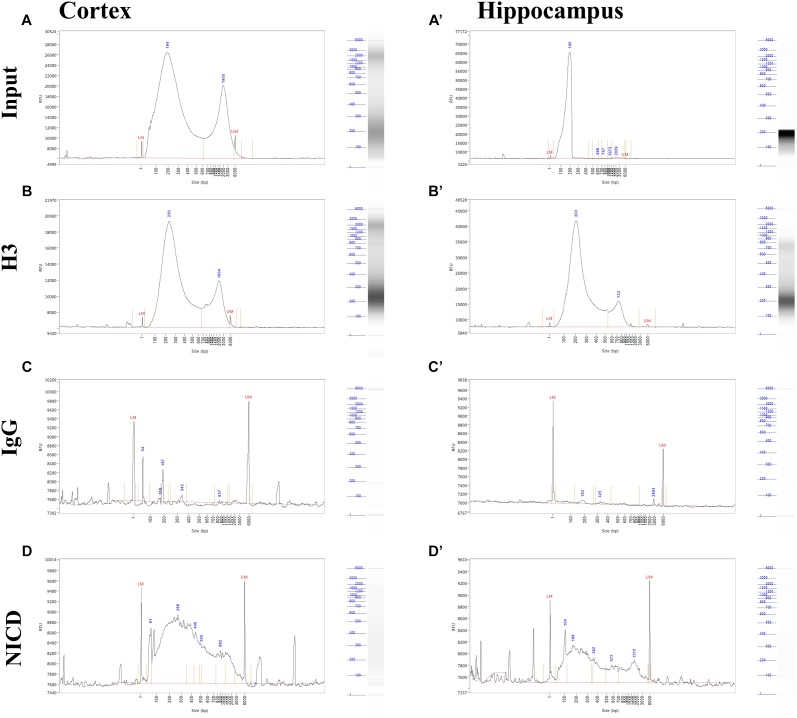
Profiling of ChIP fragments. **(A–D)** Electropherograms of automated capillary gel electrophoresis. Size profiles of purified DNA after immunoprecipitations from cortical and hippocampal CA field lysates **(A’–D’)** show the fragment length distribution for **(A, A’)** input, **(B, B’)** H3, and **(C, C’)** NICD showing a bimodal distribution and a major peak around 200 bp. **(D, D’)** IgG fragment analysis shows a sparse, mostly background peaks. DNA size (bp) is shown.

Shearing was also tested after ChIP by the mean of Fragment Analyzer. We found some inconsistencies between agarose gel and Fragment Analyzer analysis. In both cortex and hippocampus, it is possible to notice a bimodal distribution in the input as well as in immunoprecipitates (Figure 3). This may be due to the higher sensitivity of capillary gel electrophoresis to residual contaminants (ions, SDS, proteins, etc.), due to overloading or to conformation/spatial structure of DNA molecules. However, the low peak is predominant in all sample, and the majority of DNA fragments ranging between 200 and 500 bp (Figure 3A,B,D). It is also possible to notice that H3 and NICD antibodies show the same behavior in the two tissues (Figure 3B,B’,D,D’) whereas the mock precipitated IgG sample (the negative control) has barely precipitate fragments (Figure 3C,C’).

### PCR Results Analysis

**FIGURE 4:**
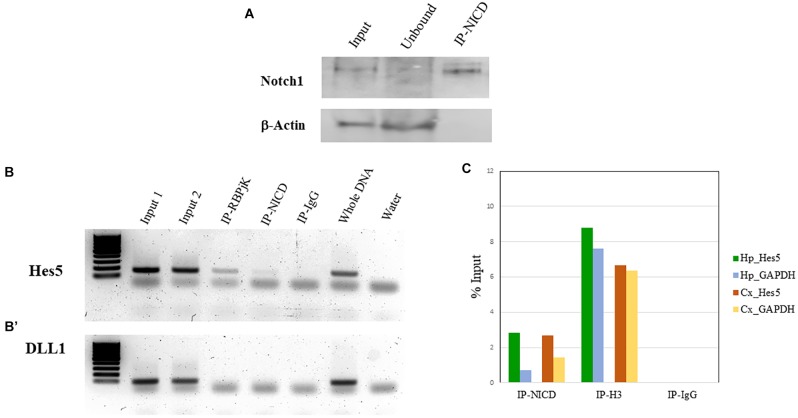
ChIP validation. **(A)** Representative western blot of Notch1 protein present in the input, enriched in the ChIP-NICD sample and absent in the unbound solute. β-Actin is used to detect the housekeeping protein in the input and unbound fractions. **(B, B’)** PCR analysis of hippocampus ChIPed with antibodies specific for RBPjK and NICD show an amplicon band for the promoter region of the canonical Notch target, Hes5 **(B)**, but no band for the promoter region of DLL1 **(B’)**, chosen as a negative control. Inputs and whole DNA are shown as a positive reference and water controls for contaminants. **(C)** Results from real-time qPCR of ChIPed tissue with the NICD antibody shows a fourfold and twofold enrichment of the specific Notch target, Hes5, compared to the GAPDH promoter region in the hippocampus and cortex, respectively. The ChIPed samples with the antibody against acetyl-histone H3 antibodies show the expected amplification of the Hes5 and the GAPDH promoter in both tissues and there is no amplification in the ChIPed samples with IgG.

To validate the enrichment of NICD in the ChIP-NICD complex compared to the input and unbound fractions, we performed western blot analysis for Notch1 (c-terminus) and used β-actin as negative control. The ChIP-NICD fraction is enriched in cleaved Notch1 1.6-fold compared to the input. Precipitation is specific to NICD as indicated by the absence of β-actin, which is instead present in the input and unbound fraction (Figure 4A).

Downstream assays to investigate protein–DNA interaction at genomic binding sites are polymerase chain reaction (PCR), quantitative polymerase chain reaction (qPCR), DNA microarray (ChIP-on-chip), and massively parallel DNA sequencing (ChIP-seq).

Normal PCR is the easiest but less reliable approach to analyze a ChIP experiment product. It is a fast and cheap method to assess the quality/purity of the immunoprecipitated chromatin, but it is less employed for detection of potential target genes because it is not quantitative. In that case, it is preferable to use qPCR instead since it provides an accurate determination of levels of specific DNA in ChIP-ed samples. In both cases, primers should be designed for a region the TF binds to, the positive control, and for a region, your TF is supposed not to bind, representing the negative control. If there is previous knowledge, it is recommended to first identify the binding site sequence *in silico* in order to accurately amplify the extracted DNA.

If there are no known sites but candidate target genes are available, it is possible to design primers along the length of potential regulatory regions such as promoters and conserved noncoding sequences within intergenic regions. If candidate target genes or potential sites are not available, ChIP-chip (Buck and Lieb, 2004) or ChIP-seq (Fanelli et al., 2011; Furey, 2012) should be considered instead.

In our case, we know that RBPjK-binding motif is CGTGGGAA (Tun et al., 1994). Therefore, we designed a set of primers looking at the presence of these eight nucleotides strings within the sequence of the Hes5 promoter, a canonical Notch signaling target gene. We also chose Delta-like protein 1 (DLL1) promoter as a negative control region since there are no RBPjK-binding motifs. A good example of purity was achieved as demonstrated by the PCR and qPCR results (Figure 4). For Hes5 PCR results, we observe amplification in both RBPjK and NICD samples while there is no amplification in IgG lane suggesting that non-specific binding is absent (Figure 4B).

On the other hand, the DLL1 PCR shows no amplification (Figure 4B’), indicating the specificity of the ChIP for RBPjK and NICD targets.

Quantitative polymerase chain reaction analysis further confirms the specificity obtained using Hes5 as a positive control and GADPH as a negative control (Figure 4C). Binding efficiency is expressed by the percent input method that includes normalization for both background levels and input chromatin (Lacazette, 2016).

This protocol is suitable for sequencing (data not shown). From the ChIPed fragments, a library is prepared according to manufacturer’s instruction and loaded into the flow-cell for sequencing; trimmed sequence reads are mapped to a reference genome. Next, peaks are found using peak-calling algorithms (Nakato and Shirahige, 2017). To further analyze the data, differential binding or motif analyses are common endpoints of ChIP-seq workflows. The ChIP-Seq procedure allows to map TF-target sequences and provide a functional readout of gene activation in complex tissues, such as the brain, and under different experimental conditions: i.e., environmental enrichment, aging, neurodegeneration, neuroinflammation, etc. Understanding the downstream targets of signaling pathways critical for plasticity and neurodegeneration will facilitate the discrimination of beneficial versus detrimental genes’ products that can be employed for developing therapeutic strategies targeting brain diseases.

Concluding, there are already other protocols describing ChIP procedures (Table 1; Braveman et al., 2004; Nelson et al., 2006; Dahl and Collas, 2008; Sailaja et al., 2012; Schmidl et al., 2015). However, all these methods are either centered on abundant histone modification or use cells suspensions as starting material. The added value of this protocol is the possibility to perform all the downstream analyses described (i) from a tissue subregion, (ii) precipitating for a rare transcriptional activator (NICD), and (iii) yielding a sufficient amount of DNA with reduced background. The advantage of performing ChIP for NICD, compared to RBPJK, has undisputed advantages as only activated loci under specific conditions will be captured within the tissue revealing active transcriptional targets.

**Table 1:** Systematic comparisons of ChIP protocols from tissue.

Protocol	ChIP on hippocampus	ChIP on brain tissue	Low-cell ChIP	FastChIP	ChIPmentation	TF-ChIP
*Publication title*	Chromatin immunoprecipitation in mouse hippocampal cells and tissues.	Chromatin immunoprecipitation technique for study of transcriptional dysregulation in intact mouse brain.	A rapid micro chromatin immunoprecipitation assay (μChIP).	Protocol for the fast chromatin immunoprecipitation (ChIP) method.	ChIPmentation: fast, robust, low-input ChIP-seq for histones and TFs.	TF-ChIP method for tissue-specific gene targets.
*Reference*	Sailaja et al. (2012)	Braveman et al. (2004)	Dahl and Collas (2008)	Nelson et al. (2006)	Schmidl et al. (2015)	This study.
*Starting material used*	Mouse whole hippocampus/dissociated hippocampal cells.	One mouse brain hemisphere not perfused.	1K-100 cells and c.ca 1 mm^3^ fresh- or frozen-tissue biopsies.	Best results with 2 M cells per IP.	10 K cells for histone markers 100 K for TF.	30 mg of tissue (CA1 fields from two hippocampi or bilateral cortex).
*Protocol*	General ChIP protocol with Sepharose A/G beads.	Upstate Biotechnology ChIP assay kit for cultured cells adapted to intact mouse brain tissue.	General ChIP protocol using *ab initio* antibody–magnetic bead complexes preparation.	Fast protocol since it shortens two steps: (i) immunoprecipitation time (accelerated by the means of ultrasonic bath) (ii) cross-link reversal and DNA isolation [using a resin-based (Chelex-100)] DNA isolation procedure.	Simple ChIP protocol combined with sequencing library preparation by Tn5 transposase (“tagmentation”).	TF-ChIP protocol with Sepharose A/G beads optimized for pure tissue-specific output.
*Histone modification tested*	Not specified	Not tested	H3K9ac, H3K9m2, H3K9m3, H3K27m3, H3K9m3, H3K4m2, H3K4m3.	H3K9Ac, H3K4me2, H3K4m3, H3K27m3.	H3K4me1, H3K4me3, H3K27ac, H3K27me3, H3K36me3.	Not tested.
*TF tested*	Not specified	Sp1	RNAPII	RNA polymerase II, hnRNP K, TBP, CREB and CBP.	CTCF, GATA1, PU.1, and REST.	NICD and RBPjK.
*Time estimated*	3 d	2–3 d	1 d	4–6 h	2–3 d	2 d
*Advantages*	Optimized for sparsely populated tissues like the brain.	Optimized for sparsely populated tissues like the brain.	(i) Fast procedure; (ii) a small amount of starting material required.	Very fast.	(i) Fast; (ii) low cell numbers for histone marks and relatively low cell numbers for TFs.	(i) Allow detection of dynamic bindings; (ii) tissue-specific; (ii) optimized for low-abundance or not-direct DNA-binding proteins.
*Drawbacks*	(i) Needs to be optimized depending on applications; (ii) lack of specific information about TF or histone modification.	(i) ChIP kit needs to be used; (ii) uncertain performance for low-abundance or not-direct DNA-binding proteins.	(i) Not tested for sparsely populated tissues like the brain; (ii) uncertain performance for low-abundance or not-direct DNA-binding proteins; (iii) small yield. Not suitable for analysis of more than one genomic locus.	(i) Not tested for sparsely populated tissues like the brain; (ii) uncertain performance for low-abundance or not-direct DNA-binding proteins.	(i) Additional reagent requirements (transposase); (ii) not tested for tissues; (iii) uncertain performance for low-abundance or not-direct DNA-binding proteins.	Optimization required depending on the tissue density and the TF studied.
Advancements with the present protocol.	(i) The antibody for TF has been clearly stated and referenced; (ii) the application has been specified.	(i) Ideally suited for any ChIP-grade antibody, no need for an expensive kit; (ii) designed for co-TF.	(i) Tested and validated for sparsely populated tissue; (ii) designed for co-TF; (iii) high yield from small micro-dissected tissue.	(i) Tested and validated for sparsely populated tissue; (ii) high yield from small micro-dissected tissue.	(i) All reagents are listed; (ii) tested and validated for tissue but also suitable for cell suspension; (iii) high yield from small micro-dissected tissue.	(i) optimized for low expressed-TF-ChIP; (ii) tested and validated to obtain high yields of DNA from sparsely dense tissue; (iii) customizable.


### Troubleshooting

Table 2 indicates issues which can occur during the procedure with a possible reason and suggestions to solve and avoid them.

**Table 2:** Troubleshooting table.

Problem	Possible reason	Solution
Fragmented chromatin concentration is too low	1. Incomplete tissue lysis. 2. Not enough tissue used for the chromatin preparation.	1. Check for single-cell suspension using a microscope. 2. If incomplete tissue lysis is excluded, try increasing the starting material amount.
Fragments are too large when visualized in Agarose gel.	1. Samples may have been over-crosslinked. 2. Too much input material was processed. 3. Insufficient sonication.	1. Reduce the crosslinking time. 2. Reduce the amount of tissues per sonication or dilute it more in Lysis buffer. 3. Conduct a sonication time course.
Fragments are too short when visualized in Agarose gel.	Samples may have been subjected to excessive shearing by sonication and/or conditions are too harsh.	Conduct a sonication time course. Do not increase the duration of sonication steps, as this could overheat the sample and lead to loss of epitopes.
DNA pellets do not solubilize after extraction.	DNA pellet is overdried.	Heat the pellet at 37°C until it is completely solubilized. Try to avoid excessive drying of the DNA pellet.
No product or a very little band for the input after PCR reactions.	1. DNA added to the PCR reaction was not enough. 2. Conditions are not optimal.	1. Add more DNA to the PCR reaction or increase the number of amplification cycles. 2. Optimize the PCR conditions for experimental primer set using purified DNA from cross-linked and fragmented chromatin.
No product in the positive control after PCR reactions.	1. Not enough chromatin or antibody added to the IP reaction. 2. DNA could have been lost during washes. 3. Incomplete elution of chromatin from beads.	1. Try to add more chromatin or more antibody. 2. Prepare new wash solutions. 3. Try increasing elution time and shake tubes more frequently.
Visible product in the negative control.	1. Insufficient washes. 2. Too much chromatin added to the IP reaction.	1. Increase the number and/or stringency of the washes after immunoprecipitation. 2. Reduce the amount of negative control antibody or the amount of chromatin.
No band for the IP product after PCR reaction.	1. Not enough antibody added to the IP reaction. 2. Not enough DNA added to the PCR reaction. 3. Not enough chromatin added to the IP reaction.	1. Typically a range of 1–5 μg of antibody is added to the IP reaction; however, the exact amount depends greatly on the individual antibody. Try to use ChIP-grade antibodies. 2. Increase the number of amplification cycles or add more DNA to the PCR reaction. 3. Try to add more chromatin or increase the amount of starting tissue.


## Data Availability

The datasets generated for this study are available on request to the corresponding author.

## Ethics Statement

Animal protocol n. 2016_32_FR released by the cantonal Veterinary office of Fribourg.

## Author Contributions

AP conducted the experiments and wrote the manuscript. LA directed the experimentation and co-wrote the manuscript.

## Conflict of Interest Statement

The authors declare that the research was conducted in the absence of any commercial or financial relationships that could be construed as a potential conflict of interest.
